# 1-(4-Fluoro­phen­yl)-5-(4-methoxy­phen­yl)pyrazolidin-3-one

**DOI:** 10.1107/S1600536809007144

**Published:** 2009-03-06

**Authors:** Bao-Jiang Dai, Yuan-Yuan Liu, Qing-Bing Xu, Jing Hu, Hong-Jun Zhu

**Affiliations:** a Nantong Jiangshan Agrochemical & Chemicals Co Ltd, Nantong 226006, People’s Republic of China; b Department of Applied Chemistry, College of Science, Nanjing University of Technology, Nanjing 210009, People’s Republic of China

## Abstract

In the mol­ecule of the title compound, C_16_H_15_FN_2_O_2_, the benzene rings are oriented at a dihedral angle of 88.61 (3)°. The five-membered ring adopts an envelope conformation. Intra­molecular C—H⋯N hydrogen bonds result in the formation of two planar five-membered rings. In the crystal structure, inter­molecular N—H⋯O and C—H⋯F hydrogen bonds link the mol­ecules, forming *R*_2_^2^(8) and *R*_2_^2^(18) ring motifs. Weak C—H⋯π inter­actions may further stabilize the structure.

## Related literature

For applications of pyrazolidin-3-one, see: Prakash *et al.* (2008 ▶); Nonaka (2003 ▶); Mabuchi & Ohtsuka (1999 ▶). For a related structure, see: Liu *et al.* (2008 ▶). For bond-length data, see: Allen *et al.* (1987 ▶). For ring motifs, see: Bernstein *et al.* (1995 ▶).
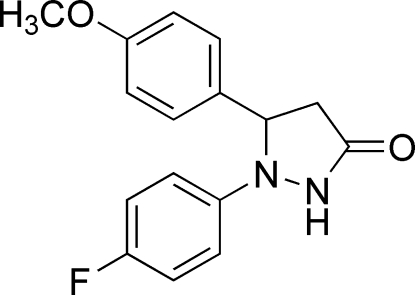


## Experimental

### 

#### Crystal data

C_16_H_15_FN_2_O_2_
*M* *_r_* = 286.30
Monoclinic, 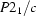

*a* = 11.455 (2) Å
*b* = 7.1590 (14) Å
*c* = 18.136 (4) Å
β = 101.05 (3)°
*V* = 1459.7 (5) Å^3^
*Z* = 4
Mo *K*α radiation
μ = 0.10 mm^−1^
*T* = 294 K
0.4 × 0.4 × 0.3 mm

#### Data collection

Enraf–Nonius CAD-4 diffractometer
Absorption correction: ψ scan (North *et al.*, 1968 ▶) *T*_min_ = 0.969, *T*_max_ = 0.991
2991 measured reflections
2844 independent reflections
1869 reflections with *I* > 2σ(*I*)
*R*_int_ = 0.023
3 standard reflections frequency: 120 min intensity decay: none

#### Refinement

*R*[*F* ^2^ > 2σ(*F* ^2^)] = 0.049
*wR*(*F* ^2^) = 0.164
*S* = 1.01
2844 reflections
191 parameters
H-atom parameters constrained
Δρ_max_ = 0.18 e Å^−3^
Δρ_min_ = −0.18 e Å^−3^

### 

Data collection: *CAD-4 Software* (Enraf–Nonius, 1985 ▶); cell refinement: *CAD-4 Software*; data reduction: *XCAD4* (Harms & Wocadlo, 1995 ▶); program(s) used to solve structure: *SHELXS97* (Sheldrick, 2008 ▶); program(s) used to refine structure: *SHELXL97* (Sheldrick, 2008 ▶); molecular graphics: *ORTEP-3 for Windows* (Farrugia, 1997 ▶) and *PLATON* (Spek, 2009 ▶); software used to prepare material for publication: *SHELXTL* (Sheldrick, 2008 ▶).

## Supplementary Material

Crystal structure: contains datablocks I, global. DOI: 10.1107/S1600536809007144/hk2631sup1.cif

Structure factors: contains datablocks I. DOI: 10.1107/S1600536809007144/hk2631Isup2.hkl

Additional supplementary materials:  crystallographic information; 3D view; checkCIF report

## Figures and Tables

**Table 1:** Hydrogen-bond geometry (Å, °)

*D*—H⋯*A*	*D*—H	H⋯*A*	*D*⋯*A*	*D*—H⋯*A*
N2—H2*A*⋯O1^i^	0.86	1.98	2.838 (2)	175
C5—H5*A*⋯N2	0.93	2.43	2.747 (3)	100
C8—H8*A*⋯F^ii^	0.97	2.45	3.388 (3)	164
C11—H11*A*⋯N1	0.93	2.53	2.885 (3)	103
C2—H2*B*⋯*Cg*2^iii^	0.93	2.71	3.589 (3)	157
C15—H15*A*⋯*Cg*1^iv^	0.93	2.89	3.801 (3)	167

Symmetry codes: (i) 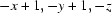; (ii) 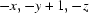; (iii) 
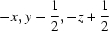
; (iv) 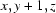. *Cg*1 and *Cg*2 are centroids of the C1–C6 andC10–C15 rings, respectively.

**Fig. 1.**
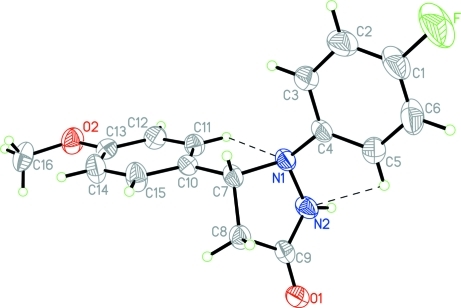
The molecular structure of the title molecule, with the atom-numbering scheme. Displacement ellipsoids are drawn at the 50% probability level. Hydrogen bonds are shown as dashed lines.

**Fig. 2.**
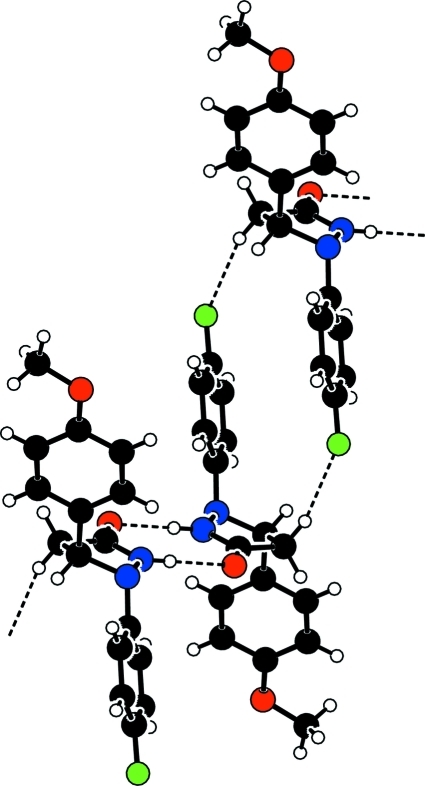
A partial packing diagram of the title compound. Hydrogen bonds are shown as dashed lines.

## supplementary crystallographic information

### Comment

Nowadays, pyrazolidin-3-one and its derivatives used as pesticide have been developed most quickly, such as antiseptic (Prakash *et al.*, 2008), insecticide (Nonaka, 2003) and herbicide (Mabuchi & Ohtsuka, 1999). We report herein the crystal structure of the title compound.

In the molecule of the title compound (Fig. 1), the bond lengths (Allen *et al.*,  1987) and angles are within normal ranges. Rings A (C1-C6) and C (C10-C15) are, of course, planar, and they are oriented at a dihedral angle of 88.61 (3)°. The five-membered ring B (N1/N2/C7-C9) adopts envelope conformation with C7 atom displaced by 0.363 (3) Å from the plane of the other ring atoms. The intramolecular C-H···N hydrogen bonds (Table 1) results in the formations of two planar five-membered rings D (N1/N2/C4/C5/H5A) and E (N1/C7/C10/C11/H11A), in which they are oriented with respect to the adjacent rings at dihedral angles of A/D = 4.87 (3) and C/E = 0.86 (3)°. So, rings C and E are coplanar, while A and D are nearly coplanar.

In the crystal structure, intermolecular N-H···O and C-H···F hydrogen bonds (Table 2) link the molecules (Fig. 2) by forming the R_2_^2^(8) and R_2_^2^(18) ring motifs (Bernstein *et al.*,  1995), in which they may be effective in the stabilization of the structure. The weak C—H···π interactions (Table 1) may further stabilize the structure.

### Experimental

The title compound was prepared according to the literature method (Liu *et al.*,  2008). Crystals suitable for X-ray analysis were obtained by dissolving the title compound (1.5 g) in ethyl acetate (25 ml) and evaporating the solvent slowly at room temperature for about 10 d.

### Refinement

H atoms were positioned geometrically, with N-H = 0.86 Å (for NH) and C-H = 0.93, 0.98, 0.97 and 0.96 Å for aromatic, methine, methylene and methyl H, respectively, and constrained to ride on their parent atoms, with U_iso_(H) = xU_eq_(C,N), where x = 1.5 for methyl H and x = 1.2 for all other H atoms.

### Crystal data

**Table:**

C_16_H_15_FN_2_O_2_	*F*(000) = 600
*M**_r_* = 286.30	*D*_x_ = 1.303 Mg m^−^^3^
Monoclinic, *P*2_1_/*c*	Mo *K*α radiation, λ = 0.71073 Å
Hall symbol: -P 2ybc	Cell parameters from 25 reflections
*a* = 11.455 (2) Å	θ = 10–13°
*b* = 7.1590 (14) Å	µ = 0.10 mm^−^^1^
*c* = 18.136 (4) Å	*T* = 294 K
β = 101.05 (3)°	Needle, colorless
*V* = 1459.7 (5) Å^3^	0.4 × 0.4 × 0.3 mm
*Z* = 4	

### Data collection

**Table:**

Enraf–Nonius CAD-4 diffractometer	1869 reflections with *I* > 2σ(*I*)
Radiation source: fine-focus sealed tube	*R*_int_ = 0.023
graphite	θ_max_ = 26.0°, θ_min_ = 1.8°
ω/2θ scans	*h* = 0→13
Absorption correction: ψ scan (North *et al.*, 1968)	*k* = 0→8
*T*_min_ = 0.969, *T*_max_ = 0.991	*l* = −21→21
2991 measured reflections	3 standard reflections every 120 min
2844 independent reflections	intensity decay: none

### Refinement

**Table:**

Refinement on *F*^2^	Secondary atom site location: difference Fourier map
Least-squares matrix: full	Hydrogen site location: inferred from neighbouring sites
*R*[*F*^2^ > 2σ(*F*^2^)] = 0.049	H-atom parameters constrained
*wR*(*F*^2^) = 0.164	*w* = 1/[σ^2^(*F*_o_^2^) + (0.1*P*)^2^] where *P* = (*F*_o_^2^ + 2*F*_c_^2^)/3
*S* = 1.01	(Δ/σ)_max_ = 0.001
2844 reflections	Δρ_max_ = 0.18 e Å^−^^3^
191 parameters	Δρ_min_ = −0.18 e Å^−^^3^
0 restraints	Extinction correction: *SHELXL97* (Sheldrick, 2008), Fc^*^=kFc[1+0.001xFc^2^λ^3^/sin(2θ)]^-1/4^
Primary atom site location: structure-invariant direct methods	Extinction coefficient: 0.038 (5)

### Special details

**Table:**

Geometry. All e.s.d.'s (except the e.s.d. in the dihedral angle between two l.s. planes) are estimated using the full covariance matrix. The cell e.s.d.'s are taken into account individually in the estimation of e.s.d.'s in distances, angles and torsion angles; correlations between e.s.d.'s in cell parameters are only used when they are defined by crystal symmetry. An approximate (isotropic) treatment of cell e.s.d.'s is used for estimating e.s.d.'s involving l.s. planes.
Refinement. Refinement of *F*^2^ against ALL reflections. The weighted *R*-factor *wR* and goodness of fit *S* are based on *F*^2^, conventional *R*-factors *R* are based on *F*, with *F* set to zero for negative *F*^2^. The threshold expression of *F*^2^ > σ(*F*^2^) is used only for calculating *R*-factors(gt) *etc*. and is not relevant to the choice of reflections for refinement. *R*-factors based on *F*^2^ are statistically about twice as large as those based on *F*, and *R*- factors based on ALL data will be even larger.

### Fractional atomic coordinates and isotropic or equivalent isotropic displacement parameters (Å^2^)

**Table:**

	*x*	*y*	*z*	*U*_iso_*/*U*_eq_	
O1	0.48784 (13)	0.2552 (2)	0.03274 (8)	0.0523 (4)	
O2	0.38835 (15)	−0.1685 (2)	−0.36578 (8)	0.0597 (5)	
N1	0.26509 (14)	0.3997 (2)	−0.11625 (9)	0.0405 (4)	
N2	0.37005 (14)	0.4183 (2)	−0.06107 (10)	0.0468 (5)	
H2A	0.4116	0.5192	−0.0556	0.056*	
F	−0.11183 (16)	0.8402 (3)	−0.07221 (14)	0.1260 (8)	
C1	−0.0185 (2)	0.7276 (4)	−0.08105 (18)	0.0724 (8)	
C2	−0.0298 (2)	0.6229 (4)	−0.14437 (16)	0.0694 (8)	
H2B	−0.0996	0.6247	−0.1803	0.083*	
C3	0.0654 (2)	0.5135 (3)	−0.15406 (13)	0.0541 (6)	
H3A	0.0603	0.4424	−0.1975	0.065*	
C4	0.16815 (17)	0.5086 (2)	−0.09998 (11)	0.0393 (5)	
C5	0.1755 (2)	0.6150 (3)	−0.03597 (12)	0.0525 (6)	
H5A	0.2443	0.6122	0.0009	0.063*	
C6	0.0806 (2)	0.7261 (4)	−0.02653 (17)	0.0723 (8)	
H6A	0.0848	0.7985	0.0165	0.087*	
C7	0.24222 (18)	0.1930 (3)	−0.12010 (11)	0.0393 (5)	
H7A	0.1569	0.1709	−0.1237	0.047*	
C8	0.30920 (19)	0.1193 (3)	−0.04418 (10)	0.0436 (5)	
H8A	0.2559	0.1040	−0.0090	0.052*	
H8B	0.3472	0.0006	−0.0501	0.052*	
C9	0.39987 (18)	0.2682 (3)	−0.01826 (11)	0.0416 (5)	
C10	0.28206 (17)	0.1019 (3)	−0.18638 (10)	0.0391 (5)	
C11	0.33591 (18)	0.1959 (3)	−0.23771 (11)	0.0443 (5)	
H11A	0.3490	0.3238	−0.2323	0.053*	
C12	0.3703 (2)	0.1032 (3)	−0.29659 (11)	0.0486 (5)	
H12A	0.4069	0.1687	−0.3302	0.058*	
C13	0.35087 (19)	−0.0874 (3)	−0.30603 (10)	0.0455 (5)	
C14	0.2963 (2)	−0.1830 (3)	−0.25569 (13)	0.0592 (7)	
H14A	0.2825	−0.3107	−0.2613	0.071*	
C15	0.2625 (2)	−0.0876 (3)	−0.19703 (12)	0.0560 (6)	
H15A	0.2254	−0.1529	−0.1636	0.067*	
C16	0.3801 (2)	−0.3669 (4)	−0.37188 (14)	0.0655 (7)	
H16A	0.4084	−0.4069	−0.4158	0.098*	
H16B	0.2987	−0.4044	−0.3758	0.098*	
H16C	0.4276	−0.4230	−0.3281	0.098*	

### Atomic displacement parameters (Å^2^)

**Table:**

	*U*^11^	*U*^22^	*U*^33^	*U*^12^	*U*^13^	*U*^23^
O1	0.0529 (9)	0.0421 (9)	0.0565 (9)	0.0060 (7)	−0.0034 (7)	0.0017 (7)
O2	0.0751 (11)	0.0584 (11)	0.0486 (9)	−0.0018 (8)	0.0194 (8)	−0.0099 (7)
N1	0.0387 (9)	0.0295 (9)	0.0521 (10)	0.0020 (7)	0.0053 (7)	−0.0001 (7)
N2	0.0361 (9)	0.0319 (9)	0.0678 (11)	−0.0014 (7)	−0.0017 (8)	0.0030 (8)
F	0.0732 (12)	0.1078 (16)	0.200 (2)	0.0392 (11)	0.0325 (13)	−0.0413 (15)
C1	0.0470 (14)	0.0575 (16)	0.114 (2)	0.0138 (12)	0.0199 (15)	−0.0134 (15)
C2	0.0464 (14)	0.0637 (17)	0.0911 (19)	0.0095 (12)	−0.0040 (13)	−0.0011 (15)
C3	0.0482 (13)	0.0478 (13)	0.0622 (14)	0.0055 (10)	−0.0001 (10)	−0.0054 (11)
C4	0.0425 (11)	0.0267 (10)	0.0486 (11)	0.0025 (8)	0.0082 (9)	0.0043 (8)
C5	0.0536 (13)	0.0472 (13)	0.0551 (13)	0.0021 (10)	0.0069 (10)	−0.0084 (10)
C6	0.0711 (18)	0.0606 (17)	0.0880 (19)	0.0055 (13)	0.0226 (15)	−0.0289 (14)
C7	0.0399 (10)	0.0281 (10)	0.0501 (11)	−0.0005 (8)	0.0090 (9)	0.0009 (8)
C8	0.0552 (13)	0.0344 (11)	0.0425 (11)	−0.0020 (9)	0.0131 (9)	0.0010 (9)
C9	0.0455 (11)	0.0328 (11)	0.0476 (11)	0.0064 (9)	0.0122 (9)	−0.0014 (9)
C10	0.0441 (11)	0.0322 (10)	0.0395 (10)	0.0001 (8)	0.0044 (8)	0.0022 (8)
C11	0.0478 (12)	0.0341 (10)	0.0495 (12)	−0.0044 (9)	0.0060 (9)	0.0027 (9)
C12	0.0550 (13)	0.0484 (13)	0.0434 (11)	−0.0081 (10)	0.0120 (10)	0.0032 (9)
C13	0.0501 (12)	0.0479 (13)	0.0365 (10)	0.0015 (10)	0.0032 (9)	−0.0026 (9)
C14	0.0896 (19)	0.0362 (12)	0.0557 (13)	−0.0053 (12)	0.0238 (13)	−0.0048 (10)
C15	0.0857 (17)	0.0367 (12)	0.0518 (12)	−0.0080 (11)	0.0287 (12)	0.0011 (9)
C16	0.0717 (16)	0.0648 (16)	0.0614 (14)	−0.0109 (13)	0.0162 (12)	−0.0278 (13)

### Geometric parameters (Å, °)

**Table:**

O1—C9	1.234 (2)	C7—C8	1.536 (3)
O2—C13	1.369 (2)	C7—C10	1.513 (3)
O2—C16	1.427 (3)	C7—H7A	0.9800
N1—N2	1.415 (2)	C8—H8A	0.9700
N1—C4	1.433 (2)	C8—H8B	0.9700
N1—C7	1.502 (2)	C9—C8	1.500 (3)
N2—C9	1.331 (2)	C10—C15	1.382 (3)
N2—H2A	0.8600	C11—C10	1.386 (3)
F—C1	1.372 (3)	C11—C12	1.378 (3)
C2—C1	1.357 (4)	C11—H11A	0.9300
C2—H2B	0.9300	C12—C13	1.388 (3)
C3—C2	1.380 (3)	C12—H12A	0.9300
C3—H3A	0.9300	C13—C14	1.382 (3)
C4—C3	1.381 (3)	C14—C15	1.381 (3)
C4—C5	1.378 (3)	C14—H14A	0.9300
C5—C6	1.384 (3)	C15—H15A	0.9300
C5—H5A	0.9300	C16—H16A	0.9600
C6—C1	1.356 (4)	C16—H16B	0.9600
C6—H6A	0.9300	C16—H16C	0.9600
			
C13—O2—C16	117.22 (18)	C7—C8—H8B	111.1
N2—N1—C4	112.97 (15)	C9—C8—C7	103.53 (16)
N2—N1—C7	104.02 (14)	C9—C8—H8A	111.1
C4—N1—C7	114.23 (15)	C9—C8—H8B	111.1
N1—N2—H2A	122.4	H8A—C8—H8B	109.0
C9—N2—N1	115.13 (16)	O1—C9—N2	125.42 (19)
C9—N2—H2A	122.4	O1—C9—C8	126.75 (18)
C2—C1—F	118.3 (3)	N2—C9—C8	107.82 (17)
C6—C1—F	118.8 (3)	C15—C10—C11	117.64 (19)
C6—C1—C2	122.9 (2)	C15—C10—C7	117.95 (18)
C1—C2—C3	118.2 (2)	C11—C10—C7	124.40 (18)
C1—C2—H2B	120.9	C10—C11—H11A	119.4
C3—C2—H2B	120.9	C12—C11—C10	121.1 (2)
C2—C3—C4	120.7 (2)	C12—C11—H11A	119.4
C2—C3—H3A	119.7	C11—C12—C13	120.42 (19)
C4—C3—H3A	119.7	C11—C12—H12A	119.8
C3—C4—N1	117.15 (18)	C13—C12—H12A	119.8
C5—C4—N1	123.33 (18)	O2—C13—C14	124.3 (2)
C5—C4—C3	119.40 (19)	O2—C13—C12	116.56 (19)
C4—C5—C6	120.0 (2)	C14—C13—C12	119.17 (19)
C4—C5—H5A	120.0	C15—C14—C13	119.6 (2)
C6—C5—H5A	120.0	C15—C14—H14A	120.2
C1—C6—C5	118.9 (2)	C13—C14—H14A	120.2
C1—C6—H6A	120.6	C10—C15—H15A	119.0
C5—C6—H6A	120.6	C14—C15—C10	122.1 (2)
N1—C7—C8	104.13 (15)	C14—C15—H15A	119.0
N1—C7—C10	112.66 (16)	O2—C16—H16A	109.5
N1—C7—H7A	109.0	O2—C16—H16B	109.5
C8—C7—H7A	109.0	O2—C16—H16C	109.5
C10—C7—C8	112.90 (16)	H16A—C16—H16B	109.5
C10—C7—H7A	109.0	H16A—C16—H16C	109.5
C7—C8—H8A	111.1	H16B—C16—H16C	109.5
			
C4—N1—N2—C9	109.31 (19)	C4—C5—C6—C1	0.1 (4)
C7—N1—N2—C9	−15.1 (2)	C5—C6—C1—C2	0.8 (5)
N2—N1—C4—C3	174.02 (17)	C5—C6—C1—F	−178.9 (3)
N2—N1—C4—C5	−2.1 (3)	N1—C7—C8—C9	−21.70 (19)
C7—N1—C4—C3	−67.3 (2)	C10—C7—C8—C9	100.83 (19)
C7—N1—C4—C5	116.5 (2)	N1—C7—C10—C11	1.1 (3)
N2—N1—C7—C8	22.13 (18)	N1—C7—C10—C15	−178.22 (19)
N2—N1—C7—C10	−100.56 (17)	C8—C7—C10—C11	−116.5 (2)
C4—N1—C7—C8	−101.47 (18)	C8—C7—C10—C15	64.2 (2)
C4—N1—C7—C10	135.84 (17)	O1—C9—C8—C7	−165.5 (2)
N1—N2—C9—O1	179.81 (18)	N2—C9—C8—C7	13.5 (2)
N1—N2—C9—C8	0.8 (2)	C7—C10—C15—C14	−179.7 (2)
C16—O2—C13—C12	173.8 (2)	C11—C10—C15—C14	0.9 (3)
C16—O2—C13—C14	−6.0 (3)	C12—C11—C10—C7	179.69 (19)
C3—C2—C1—C6	−1.5 (4)	C12—C11—C10—C15	−1.0 (3)
C3—C2—C1—F	178.3 (3)	C10—C11—C12—C13	0.5 (3)
C4—C3—C2—C1	1.2 (4)	C11—C12—C13—O2	−179.71 (19)
N1—C4—C3—C2	−176.6 (2)	C11—C12—C13—C14	0.1 (3)
C5—C4—C3—C2	−0.4 (3)	O2—C13—C14—C15	179.7 (2)
N1—C4—C5—C6	175.7 (2)	C12—C13—C14—C15	−0.1 (4)
C3—C4—C5—C6	−0.3 (3)	C13—C14—C15—C10	−0.4 (4)

### Hydrogen-bond geometry (Å, °)

**Table:**

*D*—H···*A*	*D*—H	H···*A*	*D*···*A*	*D*—H···*A*
N2—H2A···O1^i^	0.86	1.98	2.838 (2)	175
C5—H5A···N2	0.93	2.43	2.747 (3)	100
C8—H8A···F^ii^	0.97	2.45	3.388 (3)	164
C11—H11A···N1	0.93	2.53	2.885 (3)	103
C2—H2B···Cg2^iii^	0.93	2.71	3.589 (3)	157
C15—H15A···Cg1^iv^	0.93	2.89	3.801 (3)	167

Symmetry codes: (i) −*x*+1, −*y*+1, −*z*; (ii) −*x*, −*y*+1, −*z*; (iii) −*x*, *y*−1/2, −*z*+1/2; (iv) *x*, *y*+1, *z*.
